# The Methylene Blue Reaction of Russo in Enteric Fever

**Published:** 1906-09-22

**Authors:** J. D. Rolleston

**Affiliations:** Assistant Medical Officer, Grove Hospital.


### New Clinical Methods.

### THE METHYLENE BLUE REACTION OF RUSSO IN ENTERIC FEVER.

By J. D. Rolleston, M.D., Assistant Medical Officer, Grove Hospital.

Russo, of Catania, has recently introduced as a substitute for the diazo reaction a watery solution of methylene blue (1 in 1,000), for which he claims the following advantages: Simplicity of technique, durability of the solution, and greater diagnostic value. Russo's test is performed by adding 4 drops of the methylene blue solution to 4 or 5 cc. of urine. Urines which are turbid from excess of urates or phosphates should first be filtered. If the reaction is positive, a uniform mint or emerald green coloration is obtained when the test-tube is shaken. If the reaction is negative, the reagent does not change colour, or at most only a light green coloration results. Naturally between the two extremes various shades of colour obtain, as is the case in the diazo reaction.

This paper is based on observations made on 70 cases admitted to hospital certified to be suffering from enteric fever. In 54 cases this diagnosis was confirmed by clinical evidence and Widal's test. The remaining 16 were found to have other diseases. Of the latter only 4 presented a positive reaction. One was a case of carcinoma of the stomach and peritoneum, 2 were cases of infective endocarditis, and 1 was a case of lobar pneumonia. All died within a few days of admission. A positive diazo reaction was also present in these cases.* The 12 negative cases were diagnosed respectively as follows: Lobar pneumonia, 2; influenza, 2; pleurisy, 1; bronchitis, 1; appendicitis, 1; cirrhosis of liver, 1; cholelithiasis, 1; leucocythæmia, 1; erythema nodosum, 1; cerebral syphilis, 1. Of the 54 cases of enteric fever, 44 were positive, 10 were negative. With two exceptions, which were cases of moderate severity, all the latter were very mild cases or convalescent when the reaction was first tried. The following shows the two reactions in cases admitted at various stages of the disease:—

| | Methylene Blue Reaction. | | Diazo Reaction. | |
|---|---|---|---|---|
| Cases admitted in— | Positive. | Negative. | Positive. | Negative. |
| First week ... | 7 | 1 | 6 | 2 |
| Second week ... ... | 15 | 4 | 8 | 11 |
| Third week ... ... | 15 | 0 | 10 | 5 |
| Fourth week ... ... | 5 | 1 | 1 | 5 |
| Fifth week ... ... | 2 | 2 | 0 | 4 |
| Sixth week ... ... | 0 | 2 | 0 | 2 |
| Total ... ... | 44 | 10 | 25 | 29 |

The dates at which the reactions became finally negative are shown below, the fatal cases having been excluded:—

| | Methylene Blue Reaction. Cases. | Diazo Reaction. Cases. |
|---|---|---|
| First week ... ... ... ... | 0 | 0 |
| Second week ... ... ... | 4 | 7 |
| Third Week ... ... ... | 11 | 11 |
| Fourth week ... ... ... | 12 | 2 |
| Fifth week and later ... ... | 11 | 0 |
| Total ... ... ... ... ... | 38 | 20 |

* The occasional presence of the diazo reaction in these diseases is noted by Rivier (*loc. cit.*).

It is evident from the above figures that the methylene blue reaction is more constant and more persistent in enteric fever than the diazo reaction. That disappearance of Russo's reaction coincides more closely with the commencement of lysis, and that, therefore, *ceteris paribus*, a negative methylene blue reaction is a surer sign of convalescence than a negative diazo reaction is shown by the fact that in all the 38 cases in which Russo's test was employed the two events occurred in the same week; whereas in the 20 cases in which the diazo reaction was concurrently used, in 14 the two events occurred in the same week, and in 6 lysis did not commence till the week following the disappearance of the reaction.

The two reactions resemble one another in that, before disappearing entirely, there is usually a gradual diminution in their intensity, the successive grades in the methylene blue reaction being conveniently designated by the letters M B R +, M B R —, M B R — —, which correspond with shades of colour varying from a deep green in the first case to a light green in the second, and a decided blue in the third. Both reactions before becoming finally negative may be occasionally positive. Another point of resemblance between the two reactions is seen in their occasional disappearance in spite of aggravation of the general condition. This indicates the supervention of a secondary infection or impermeability of the kidneys to the colouring matter. Thus in 3 cases in which, though the two reactions had become negative, the patient was obviously worse, 1 died of lobar pneumonia, 1 of perforative peritonitis, and 1 of hæmorrhage.

On occurrence of pyrexia in convalescence from enteric fever, in the absence of enlargement of the spleen or of a fresh outbreak of rose spots or other obvious physical signs, determination of the nature of such pyrexia may be a matter of no litle difficulty. The diazo reaction is often a valuable guide, but in some cases of definite relapse it may fail to be positive. In such cases Russo's reaction is a more delicate test, since it often becomes positive again, while the diazo reaction remains negative. Thus, in 10 cases of relapse the diazo reaction which had previously become negative became positive again in 3 cases only, while Russo's reaction became positive in 7 cases. It is noteworthy that those relapses in which the diazo reaction became positive again were more severe than those in which Russo's reaction alone was positive; while in those in which both tests remained negative, the pyrexia was of shorter duration still.

As with the diazo reaction a positive result may be obtained at a very early stage of the disease before Widal's test gives definite results. In 1 of my cases a positive methylene blue reaction appeared as early as the second day, though the diazo reaction in the same case did not become positive till the fifth.

Contrary to what is stated by Russo, I have found the methylene blue reaction positive in several cases of scarlet fever, lobar and broncho-pneumonia. I have also found it occasionally, though exceptionally, present in diphtheria and serum eruptions. Like the diazo reaction it is almost always present in measles. It was positive in 18 out of 20 cases in which I employed it.

*Conclusions.*—Russo's reaction persists longer in enteric fever than the diazo reaction.[1]

The disappearance of Russo's reaction corresponds more closely with lysis than does the disappearance of the diazo reaction.[2]

In relapses, a positive Russo's reaction is more frequently found than a positive diazo reaction.[3]

The diagnostic value of Russo's reaction is similar, but not superior, to the diazo reaction.[4]

[1] Gazette des Hôpitaux, May 25, 1901. [2] Thèse de Paris, 1898. [3] Lancet, February 4, 1905. [4] Riforma Medica, May 13, 1905.

